# Classification model for accuracy and intrusion detection using machine learning approach

**DOI:** 10.7717/peerj-cs.437

**Published:** 2021-04-07

**Authors:** Arushi Agarwal, Purushottam Sharma, Mohammed Alshehri, Ahmed A. Mohamed, Osama Alfarraj

**Affiliations:** 1 Amity School of Engineering and Technology, Amity University, Uttar Pradesh, India; 2 Department of Information Technology, College of Computer and Information Sciences, Majmaah University, Majmaah, Riyadh, Saudi Arabia; 3 Department of Computer Science, College of Computer and Information Sciences, Majmaah University, Majmaah, Saudi Arabia; 4 Faculty of Computer and Information, Assiut University, Assiut, Egypt; 5 Department of Computer Science, Community College, King Saud University, Riyadh, Saudi Arabia

**Keywords:** Intrusion detection system, K-Nearest Neighbors (KNN), Support vector machine (SVM), Naive Bayes (NB), UNSWNB15 dataset

## Abstract

In today’s cyber world, the demand for the internet is increasing day by day, increasing the concern of network security. The aim of an Intrusion Detection System (IDS) is to provide approaches against many fast-growing network attacks (e.g., DDoS attack, Ransomware attack, Botnet attack, etc.), as it blocks the harmful activities occurring in the network system. In this work, three different classification machine learning algorithms—Naïve Bayes (NB), Support Vector Machine (SVM), and K-nearest neighbor (KNN)—were used to detect the accuracy and reducing the processing time of an algorithm on the UNSW-NB15 dataset and to find the best-suited algorithm which can efficiently learn the pattern of the suspicious network activities. The data gathered from the feature set comparison was then applied as input to IDS as data feeds to train the system for future intrusion behavior prediction and analysis using the best-fit algorithm chosen from the above three algorithms based on the performance metrics found. Also, the classification reports (Precision, Recall, and F1-score) and confusion matrix were generated and compared to finalize the support-validation status found throughout the testing phase of the model used in this approach.

## Introduction

Today, people across the world in their personal and professional life use several devices connected to the internet daily. This increased use of the internet has resulted in the increment of internet security threats, i.e., network attacks. These attacks are harmful for the operation of devices, as it can access the system’s authorized data (Abedin et al., 2018). An Intrusion Detection System (IDS) is designed to ensure network security from the network attacks and threats. Intrusion Detection System (IDS) provides a way for monitoring network traffic when some suspicious activities and known threats have occurred and sends up alerts after discovering such activities in the network system.

In a network, the most important and concerning parameter about one’s data is the security assurance that the crucial data will be kept safe from the third-party intrusion attackers. As technology is growing fast day by day, new mechanisms are being invented to achieve this goal. As we have already seen, the same technology that was invented to defend the cyber-space is now being used for performing security breach (Alazab et al., 2012).

For example, the first “virus” program was created to test the computer system’s efficiency. But the same technology has now evolved into one of today’s most alarming issues—ransomware attacks. As the world is witnessing new “smart” technologies like Artificial Intelligence (AI), Augmented Reality (AR) etc., the attackers are also getting smart and evolving their attack patterns by using the very same technologies. This creates the paradox situation: are these new technologies really doing what they were intended to do or doing the reverse? To ensure that technologies are put to good use they were invented for, the defense mechanism against intrusion attacks should get “smarter”. Hence, there is the need to step forward to not only the advanced technology but the smartest one. Machine learning (ML) approaches can reach this goal. With the machine learning advanced models, we can predict and classify the data-theft (Janarthanan & Zargari, 2017), intrusion behaviors, and its characteristics, which will enable us to train our network more efficiently so that it can detect intrusion attacks and predict the possible ways to mitigate it. Once the network is trained using the machine learning model, it will become difficult for intruders to bypass the security mechanisms and access the main network framework as machine learning models can quickly predict intrusions based on the training data provided (Venkatraman, Alazab & Vinayakumar, 2019). Also, if a new type of attack takes place which the model does not recognize, the model has the capability to adjust itself and provide minimum security mechanisms against it and adapt itself according to the new attack by storing the information extracted from the data (Numan et al., 2020). But the main concern remains: how to find the best-suited algorithm that should be used to train the model to classify the attacks accurately? In this work, we are focusing on this part and comparing the three most reliable classification algorithms of machine learning—Support Vector Machine (SVM), K-Nearest Neighbor (KNN) and Naïve Bayes (NB) to find out the accuracy level of each of them and the best-suited algorithm for the model. In this classification model, we assume the following:

1. The latest UNSW-NB15 dataset used to train the model about the data patterns.
2. The three classification models compared to determine the best algorithm based on accuracy, classification report, and confusion matrix.
3. The dataset will be pre-processed to prepare it for classification training.
4. The resulting classification model will predict the intrusion attacks and classify them according to their relevant classes.

An Intrusion Detection System (IDS) can be categorized into several ways described are as follows:

### Active and passive IDS

An Active IDS, aka the intrusion prevention and detection system, is designed to automatically block suspicious attacks without any interference required by a user or operator. It gives the real-time response to an attacker.

Unlike an active IDS, a passive IDS is not designed in the way of evaluating any protection or correction functions by its own. It only monitors and evaluates the network traffic activities in the system and alerts if any attacks occur.

### Network intrusion detection systems and host intrusion detection systems

NIDS consists of a network device with a Network Interface Card (NIC) operating and a separate management interface. It is configured at a single point or different strategic points inside the network, to monitor the incoming and outgoing traffic to and from all the devices in the network.

HIDS constantly monitors the incoming and outgoing packets from the network system; system files are audited, and alerts are sent to the system administrator if any threats occur in the system. It has the advantage that it does not require any hardware installation, as IDS installed inside the existing system components.

### Knowledge-based (signature-based) IDS and behavior-based (anomaly-based) IDS

Knowledge-based (signature-based) IDS sends alert messages if the attack’s signature is matched with the preconfigured and predetermined pattern stored in the database. As these patterns are updated continuously to achieve the reports of the correct threats.

Behavior-based (Anomaly-based) IDS does the regular monitoring of network traffic and compares it with the analytical model, where the analytical model consists of the bandwidth used, protocols predefined for the network traffic, and network port and devices.

### Classification algorithms

Data mining is defined as extracting or mining the information and knowledge from massive datasets. The extracted data can be used in many applications as market analysis, science exploration, production control, etc. Classification algorithms are one of the main concepts in the machine learning approach. They are used to classify unlabeled data to separate classes. The algorithms used in work are as follows:

- Support Vector Machine (SVM): Support Vector Machine (SVM) is one of the most reliable classification algorithms in machine learning because it supports a fast and simple prediction process compared to other algorithms. It classifies the data points based on the support vectors in a data repository to make a hyperplane that divides the class labels into their related classes.
- K-Nearest Neighbor (KNN): K-Nearest Neighbor (KNN) is another reliable classification algorithm used for classifying data classes. One of its promising features is that it can be used for both classification regression purposes.
- Naïve Bayes (NB): Naïve Bayes is a classification algorithm that is based on Bayes’ theorem. Here, the classifier predicts that there is no dependency among the predictors. In other words, the classifier assumes that there is no relation between one feature in a class to any other feature.

The main motivation behind our approach is to find the smartest algorithm which can keep up with the increasing threat of intrusion. As we already know, machine learning classification algorithms can be put to use to achieve this goal, we dive deep and even find the best suited machine learning algorithm which can predict intrusion by analyzing attack patterns and the data provided. This goes far deep into the process of how these algorithms accomplish this? In the coming sections, we have discussed the training and testing processes performed by the algorithms to analyze the attack patterns and provide a better accuracy result than existing models (Canbay & Sagiroglu, 2015).

Now coming to the question—why to use the proposed model over already existing models? As we have gone through the literature survey and previous works related to intrusion detection system (IDS), we found that the algorithms that were used were not providing enough accuracy results. To ensure this statement, we considered the dataset that were used in an existing model and performed the training and testing on the dataset. Then we performed our proposed model on the same dataset and compared the results. We found that, the proposed model has a better accuracy rate by approximately 10% than the existing model.

The better accuracy performs as the main parameter to deduce the performance factor of the algorithm which will be used to analyze and predict the intrusion. The better the accuracy, the better the performance of the prediction model and the better the prediction will be (Sriram et al., 2020a, 2020b). Hence, the proposed model can be considered as reliable and best suited for integrating with the model.

### Related work

Many researchers have analyzed and studied the Intrusion Detection System field, and recently more and more machine learning approaches are aligning with it to provide a better solution against intrusion.

- Ashraf, Ahmad & Ashraf (2018) proposed comparing some of the most efficient machine learning algorithms - J48, Naïve Bayes, and Random Forest. This research aimed to deduct a better detection rate and accuracy of the Intrusion Detection System. The comparison was used to draw new patterns and procedures to overcome vast amount of audit data.
- Colas & Brazdil (2006) proposed another comparative study to determine the best-suited algorithm in case of classification problems. The comparison was between Support Vector Machine (SVM) to K-Nearest Neighbor (KNN) and Naïve Bayes algorithms. Based on the performance analysis and processing time, they depicted a feature comparison between the above algorithms.
- Jiang et al. (2012) developed a text categorization model using an improved and better KNN text categorization (INNTC) and one pass clustering algorithm, which shows that the combination of the two-classification algorithm improves text categorization by reducing text redundancy better than typical KNN, Naïve Bayes and SVM algorithms.
- The one pass clustering algorithm uses least distance principle to divide the training text samples into hyper spheres and then uses KNN text categorization to employ the data classification model.
- Aljawarneh, Aldwairi & Yassein (2018) provide insight into the network traffic meta-heuristic properties to create a better intrusion detection system. While large amount of data needs to be captured for performing an efficient analysis, a better model needs to be developed to feed the data into it. This work uses the NSL KDD dataset with a ratio of 80/20 for training/testing set. A new hybrid model was developed to estimate the intrusion level threshold on the network transaction data for training. From the results, it was found that the hybrid approach has a remarkable effect on the reduction of the computational and time complexity involved. The hybrid model’s accuracy was 99.81% and 98.56% for binary and multi-class datasets, respectively. While obtaining low false and high false negative rates, the issues were handled using data filtration through Vote algorithm with information gain. The hybrid algorithm consists of several classifiers – J48, Random Tree, Naïve Bayes etc.
- Elejla et al. (2019) showed a comparison between several classification algorithms such as KNN, SVM, Decision tree, Naïve Bayes, and Neural network to predict ICMPv6 based DDoS attacks by monitoring the network traffic and behavior of the attacks. The comparison showed that KNN was the fastest among the other algorithms to detect the attacks where neural network achieved the slowest rate. The classifiers detected most of the attacks ranging from 73% to 85% of the attacks.
- Ugochukwu, Bennett & Harcourt (2018) used KDD Cup’99 dataset for the training purpose of algorithms used for the classification model. Four algorithms were performed on the dataset to find the best suited algorithm with higher accuracy: – J48, Bayes Net, Random Forest, and Random Tree. These algorithms were trained, and the accuracy level were analyzed based on how accurately they classify the various attack entries in the dataset to their class labels. The results gathered showed that the Random Forest and Random Tree algorithms were the best suited algorithms on the test dataset. The classification method was performed by finding the correlation between the dataset’s feature set using the WEKA tool and based on Precision, Recall, and F-measure using a Best First Search method.

### Technology description

The following technologies were used to implement the proposed concept:

- Data mining and probability to forecast outcomes are the features which are used by predictive modeling. Each model made up of several predictors; these are mostly to influence further results by variables.
- A statistical model made when data has been collected for specific predictors. The model may employ a simple linear equation or a complex neural network, mapped out by sophisticated software. The statistical analysis model is validated as soon as additional data is available.
- Machine learning is a branch of Artificial Intelligence (AI) technology based on generating a virtual framework to train a predictive model for improved classification accuracy.
- Traditional machine learning algorithms are:
- SVM (Support Vector Machine);
- KNN (K-Nearest Neighbor);
- NB (Naïve Bayes);
- LR (Logistic Regression);
- Apriori, RF (Random Forest), etc.

### Classification algorithms

Classification algorithms are one of the main concepts in the machine learning approach.

They are used to classify unlabeled data to separate classes. Once the data is separated to specific classes, “labels” are assigned to improve the classification quality (Sainis, Srivastava & Singh, 2018). Classifiers can be of two types:

- Binary classifiers: Where the data is separated using an either-or relation. For example, either this mail is spam or not spam.
- Multi-class classifiers: Where the data classified with more than two classes, for example, type of soil.

Some of the most reliable classification algorithms are Support Vector Machine (SVM), K-Nearest Neighbor (KNN), Naïve Bayes (NB), Random Forest (RF) etc. In our work, we compared the first three algorithms to detect the best suitable one for the model.

### Support vector machine

- Support Vector Machine (SVM) is the most reliable classification algorithm in machine learning because it supports fast and simple prediction processes compared to other algorithms. It classifies the data points based on the support vectors in a data repository to make a hyperplane that divides the class labels into their related classes.
- SVM classifies the support vectors based on the “gamma” value given to the algorithm. For example, if gamma = 0, SVM predicts the hyperplane as a curvature. If gamma = auto, SVM simply predicts the hyperplane according to the data input given.

**Figure 1:**
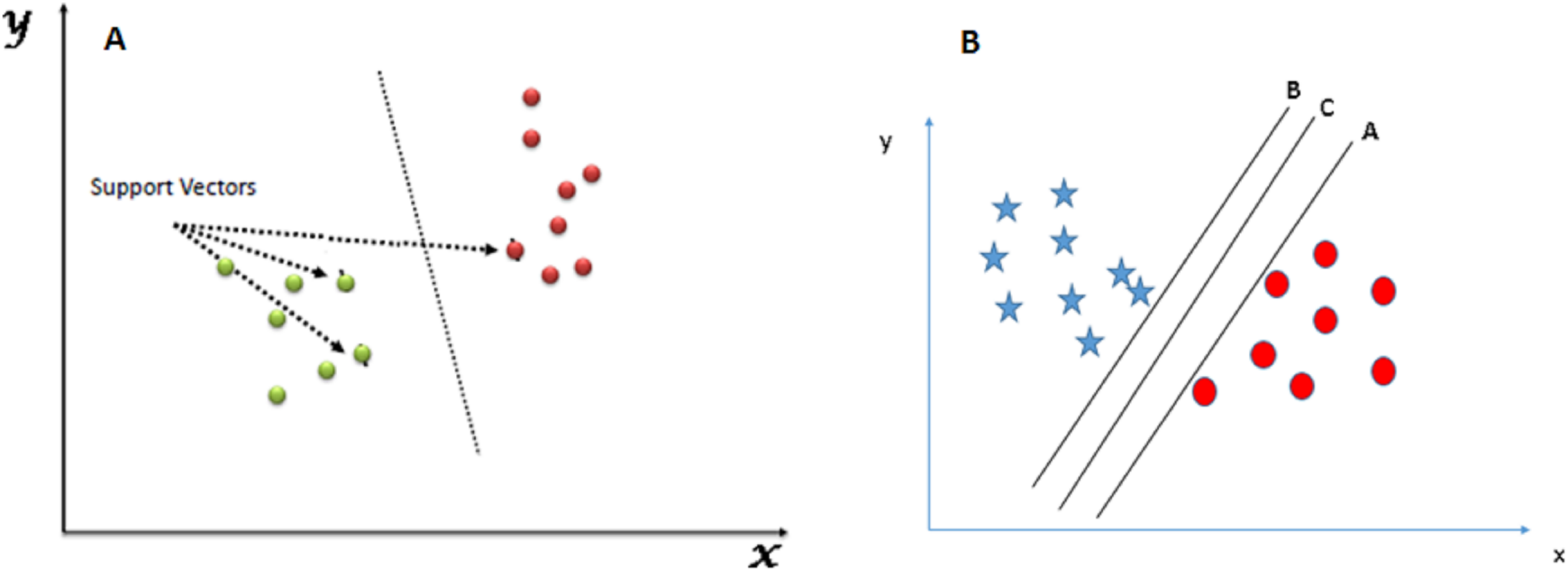
SVM classifier. (A) SVM classification technique. (B) SVM hyperplane selection.

Figure 1 shows an example of the SVM classification technique:

The following diagram shows a data repository of blue stars and red circles where we need to find out the right hyperplane to classify them accurately. Here, we have three hyperplanes A, B, and C. But the actual hyperplane that we will consider is the C hyperplane as it has the same distance from both the data points.

### K-Nearest Neighbor

- K-Nearest Neighbor (KNN) is another reliable classification algorithm mainly used for classifying data classes. One of its promising features is that it can be used for both classification regression purposes.
- The three important aspects of the KNN algorithm are as follows:
- Ease to generate the output,
- Calculation time,
- Predictive power.

**Figure 2:**
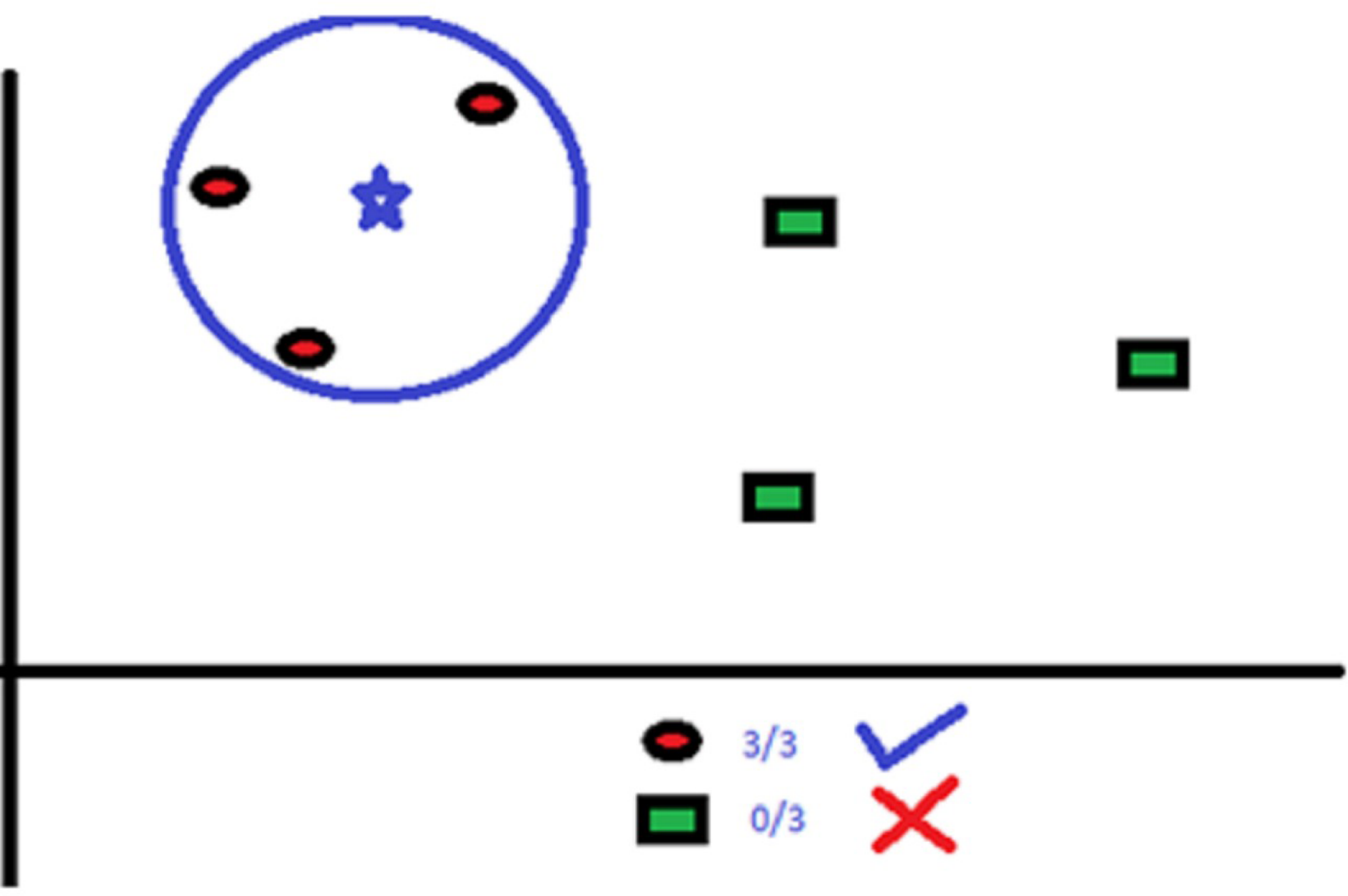
Classification with KNN where *K* = 3.

KNN uses “several neighbor” concept to classify the data points. The “K” in KNN refers to the number of neighbors to be identified. For example, in Fig. 2, there are red circles and green squares that need to be classified accurately.

Here, *K* = 3. So, the algorithm considers the classification area such that the blue star will have three red circle neighbors.

The classification area consists of no more or no less than three neighbors as *K*’s value is defined as three. We can increase or decrease the value of *K* as per the data points.

### Naïve Bayes

- Naïve Bayes is another classification algorithm which is based on Bayes’ theorem. Here, the classifier predicts that there is no dependency among the predictors. In other words, the classifier assumes that there is no relation between one feature in a class to any other feature.
- For example, if some fruit is 3 inches in diameter, red, and round then it is assumed to be an apple. But these three features individually can contribute to the prediction that the fruit is an apple. That is why this is called “Naïve”.

### Material, terms description, and tool

#### Dataset

- The dataset that we have used for this work is the UNSW-NB15 dataset of the intrusion detection system (IDS) available from AARNet systems (https://cloudstor.aarnet.edu.au/plus/index.php/s/2DhnLGDdEECo4ys).
- This dataset was generated by the IXIA PerfectStorm tool in the Cyber Range Lab of the Australian Centre for Cyber Security (ACCS). The TCPdump tool used to obtain the network traffic of 100 GB.
- This dataset contains the recent intrusion attacks and their feature sets in a labeled manner, which accurately trains the model. The feature sets extracted and stored in a separate file “UNSW-NB15 features.csv”.
- The dataset contains almost 2,000,000 records of intrusion behaviors with their parameters labeled in a particular class. Hence, this dataset selected for training our model using the best-suited algorithm.

Table 1 below shows the attack classes and subcategories with the number of samples extracted from the UNSW-NB15 dataset:

**Table 1:** Attack types classification of UNSW-NB15.

Sl. No.	Attack class	No. of samples	Attack subcategory
1.	Fuzzers	5,052	FTP, HTTP, RIP, Syslog, PPTP, FTP, DCERPC, OSPF
2.	Reconnaissance	1,760	Telnet, SNMP, NetBIOS, DNS, SCTP, MSSOL, SMTP
3.	Shellcode	224	FreeBSD, HP-UX, NetBSD, AIX, Scolnix, decoders, IRIX, MAOSX, BSDi, Solaris
4.	Analysis	527	HTML, Port Scanner, Spam
5.	Backdoors	535	Backdoors
6.	DoS	1,168	Ethernet, VPN, IRC, DP, TCP, VNC, XINETP, NTP, Asterisk, RTSP, CUPS, Cisco Skinny
7.	Worms	25	Worms
8.	Generic	7,523	SIP, IXIA, Superflow, TETP, HTTP
9.	Exploits	5,410	Evasions, SCCP, WINS, DCERPC, Dameware, SCADA, VNC, CDAP, RTSP, LPD, RDesktop, NNTP, SMB, Evasions, RADIUS, SCCP, SIP, PPTP

### Terms description

#### Confusion matrix

**Figure 3:**
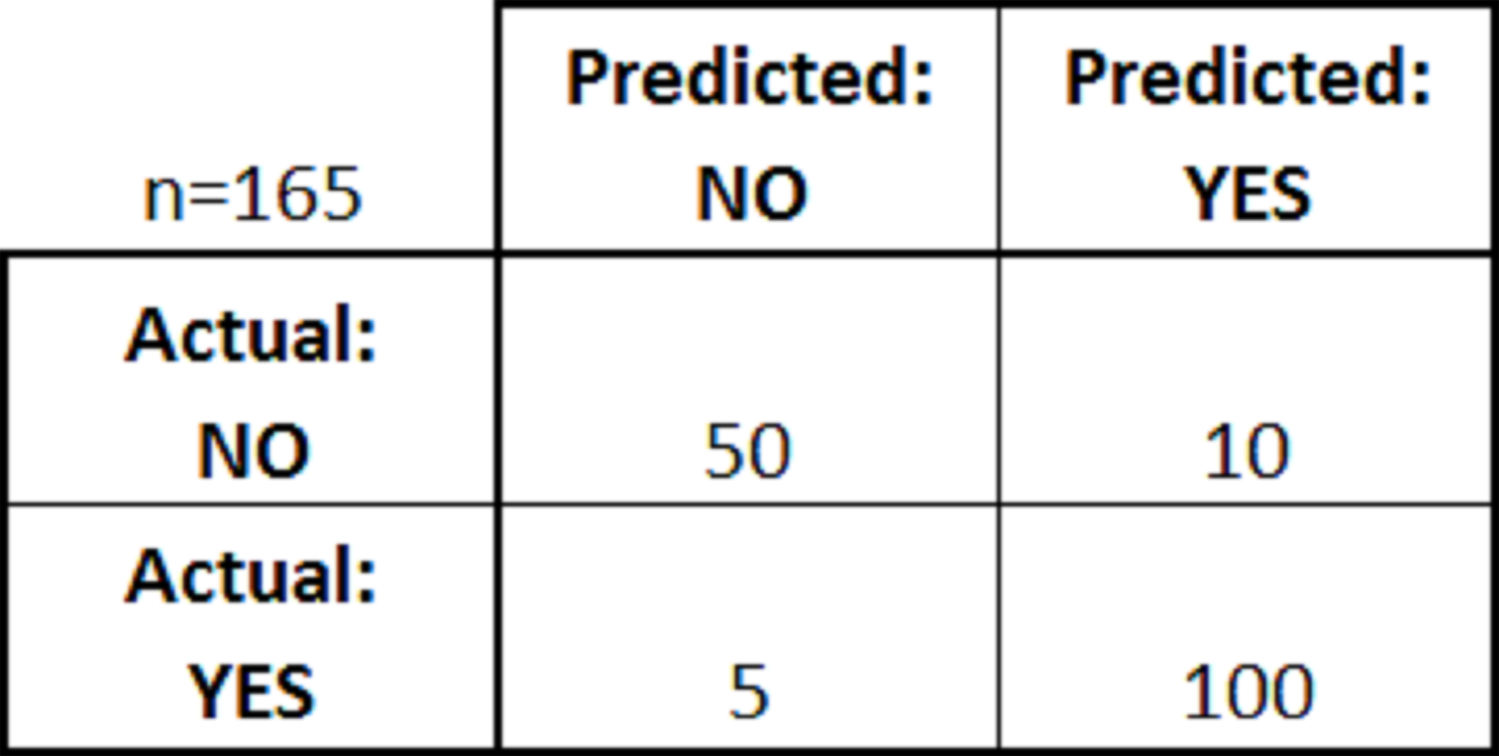
Confusion matrix.

The confusion matrix is a table set generated after the model fitting into the classification algorithm, which shows classification model’s performance activity created on a set of test data where the true values (actual values) are known (Dhanabal & Shantharajah, 2015). For example, Fig. 3 shows a confusion matrix for a simple binary classifier:

From the above figure, we can see the predicted results’ values and actual results after the classification. There are four rules in a confusion matrix followed:

- True positives (TP): In this scenario, the predicted result, and the actual result both become positive. For example, a patient is predicted to have cancer and after check-up it was found that the patient had cancer.
- True negatives (TN): In this scenario, the predicted result, and the actual result both become negative. For example, a patient did not had cancer and after check-up, it was found that the patient did not had cancer.
- False positives (FP): In this scenario, the predicted result was “yes” but the actual result was found “no”. For example, it was predicted that the patient had cancer and after check-up it was found that the patient did not had cancer.
- False negatives (FN): In this scenario, the predicted result was “no”, but the actual result was found “yes”. For example, it was predicted that the patient did not have cancer, and after check-up, it was found that the patient had cancer.

**Figure 4:**
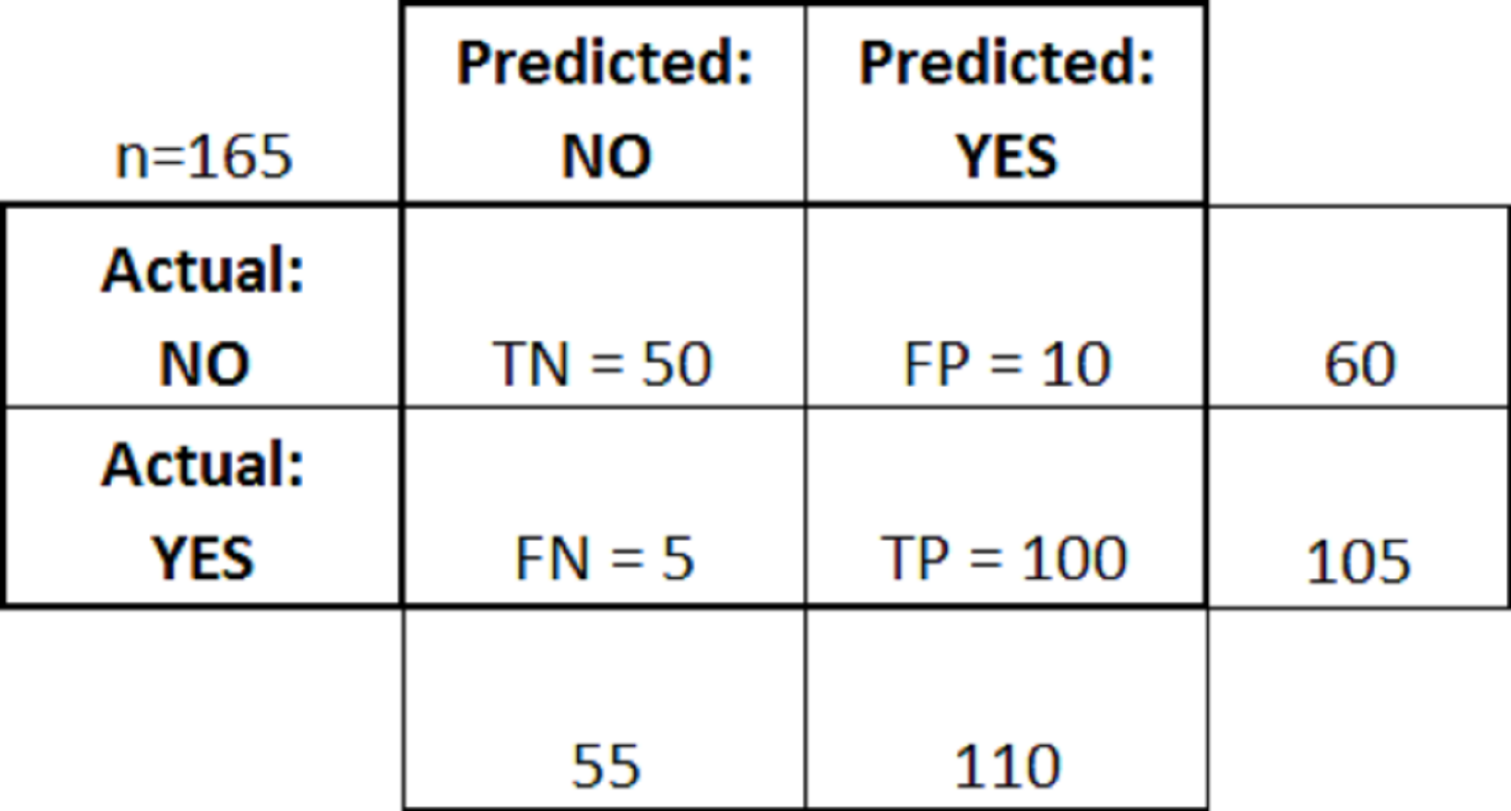
Confusion matrix with rules.

Figure 4 above shows the confusion matrix after applying the above rules.

### Classification report

The classification report is a visualization draft that displays the four base-parameters of a classification model—Precision, Recall, F1-score, and Support to generate the accuracy level because of model fitting. It helps in more straightforward interpretation and detection by integrating numerical scores with the help of a color-coded heat-map.

#### Accuracy

Accuracy is the most important performance metric that defines how capable the classification model (classifier) is. It means how accurately the algorithm is learning the data patterns in the dataset and how accurately it can predict unseen data.

#### Precision

Precision is an important performance metric that needs to be considered. It is the ratio of correctly observed positive results to all observed positive results.

#### Recall

The recall is the ratio of correctly observed positive results to the total observations in a class. It gives the result as the ratio of positive observations.

#### F1-score

F1-score is an important performance metric to consider. In some cases, F1-score has more importance than accuracy. Sometimes in a large dataset, the cost of false positives and false negatives are not the same. When they are the same, accuracy is a better option. But, when they are not the same, we need to investigate F1-score.

#### Support

Support is the number of true results observed that occurs in a class. It indicates the number of true results where predicted and actual results are the same.

### Proposed framework with implementation work

**Figure 5:**
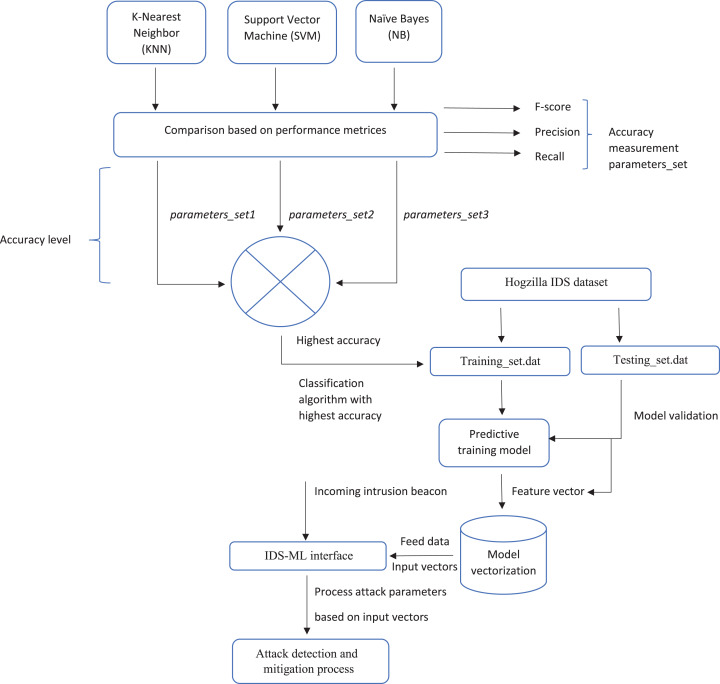
Proposed framework model.

Figure 5 below shows a summary of the proposed framework.

**Figure 6:**
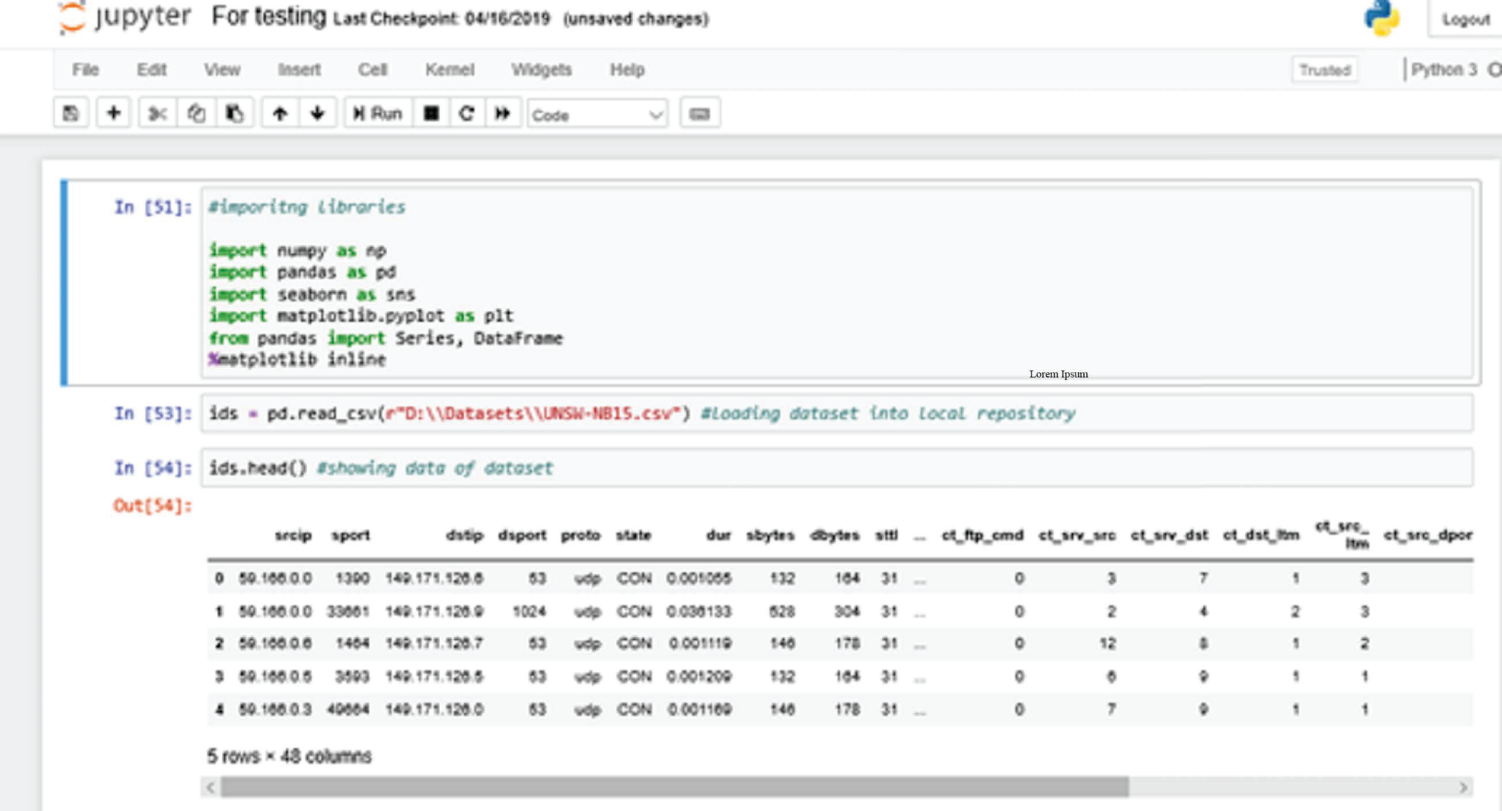
Importing libraries.

Figure 6 shows the process of importing the dataset into the local repository. At first, we are calling all the necessary library packages like numpy, pandas, seaborn etc. Using “Pandas”, we import UNSW-NB15 dataset from our local directory to the local repository of the Anaconda environment.

The ids.head() command shows data currently loaded into the local repository of the Anaconda Jupyter environment for further training purposes (Muda et al., 2011).

Here, “ids” is the variable or the DataFrame used to store the information about the dataset that has been loaded.

We are also using a “magic function” - %matplotlib inline. A magic function has the advantage that it is not necessary to invoke the function explicitly (Khan et al., 2019). Python does the work for it. The function “matplotlib inline” is used to create automatic aligned visual graphics that are not needed to be defined explicitly.

**Figure 7:**
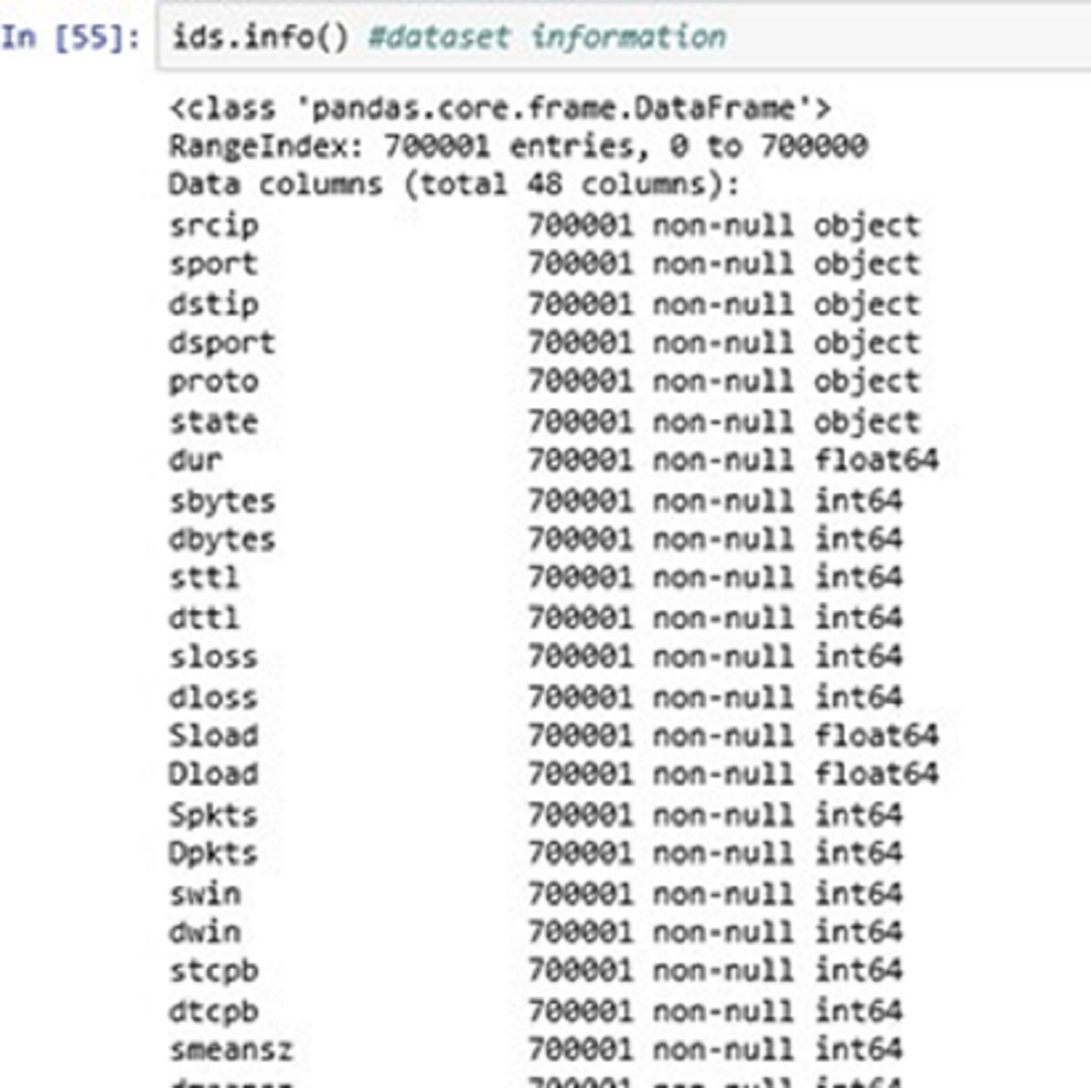
Dataset information.

Figure 7 above shows the DataFrame information of the input data. It shows the information about the dataset entries like datatypes, range index, column description etc.

**Figure 8:**
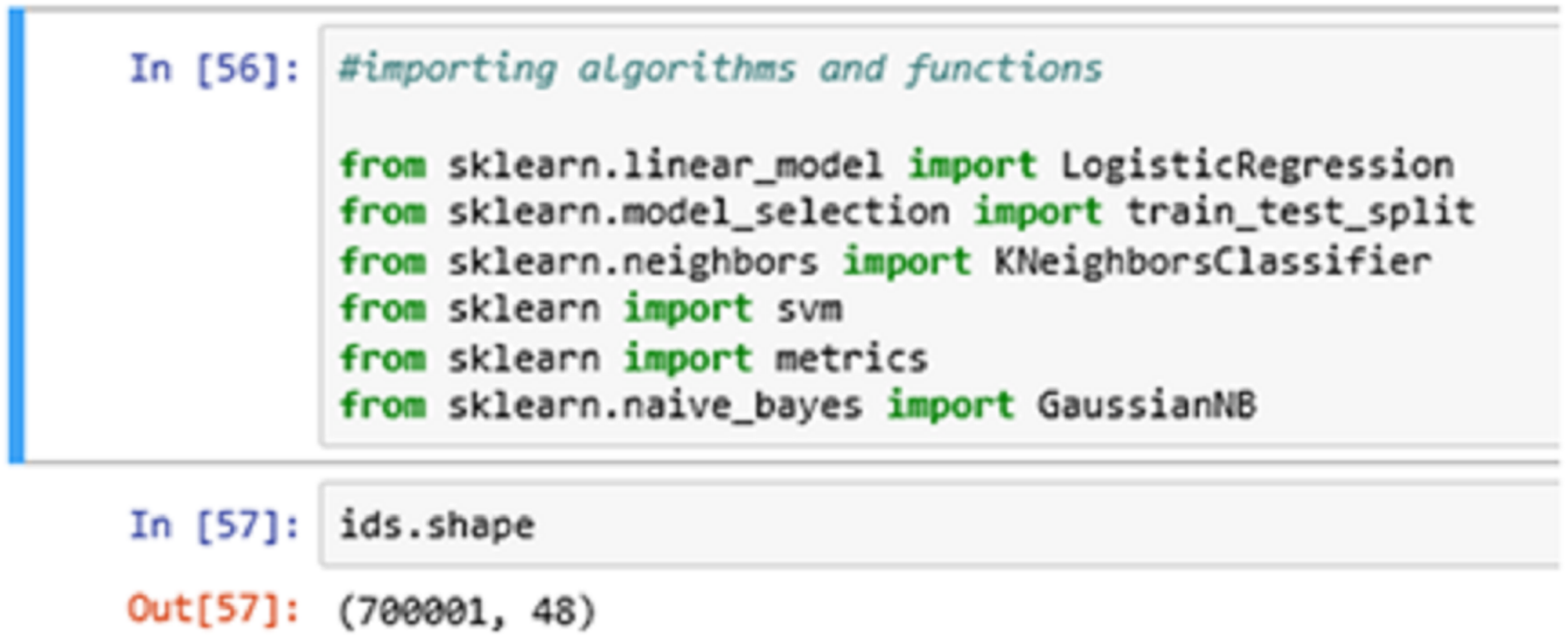
Importing algorithms.

In Fig. 8 above, we import all the necessary algorithms and other essential library packages to apply on the classification model. We are using the Scikit-learn library package to import all the necessary functions and algorithms. The ids.shape command showing the (row x column) information of the entry dataset. Here, we have 700001-row entries and 48 column entries.

A heat-map shows all the data entries of an input dataset according to their class label. This is useful when the model used for the training purposes is a classification model. The classifier can easily distinguish between the class labels using the heat-map (Shah & Issac, 2018).

**Figure 9:**
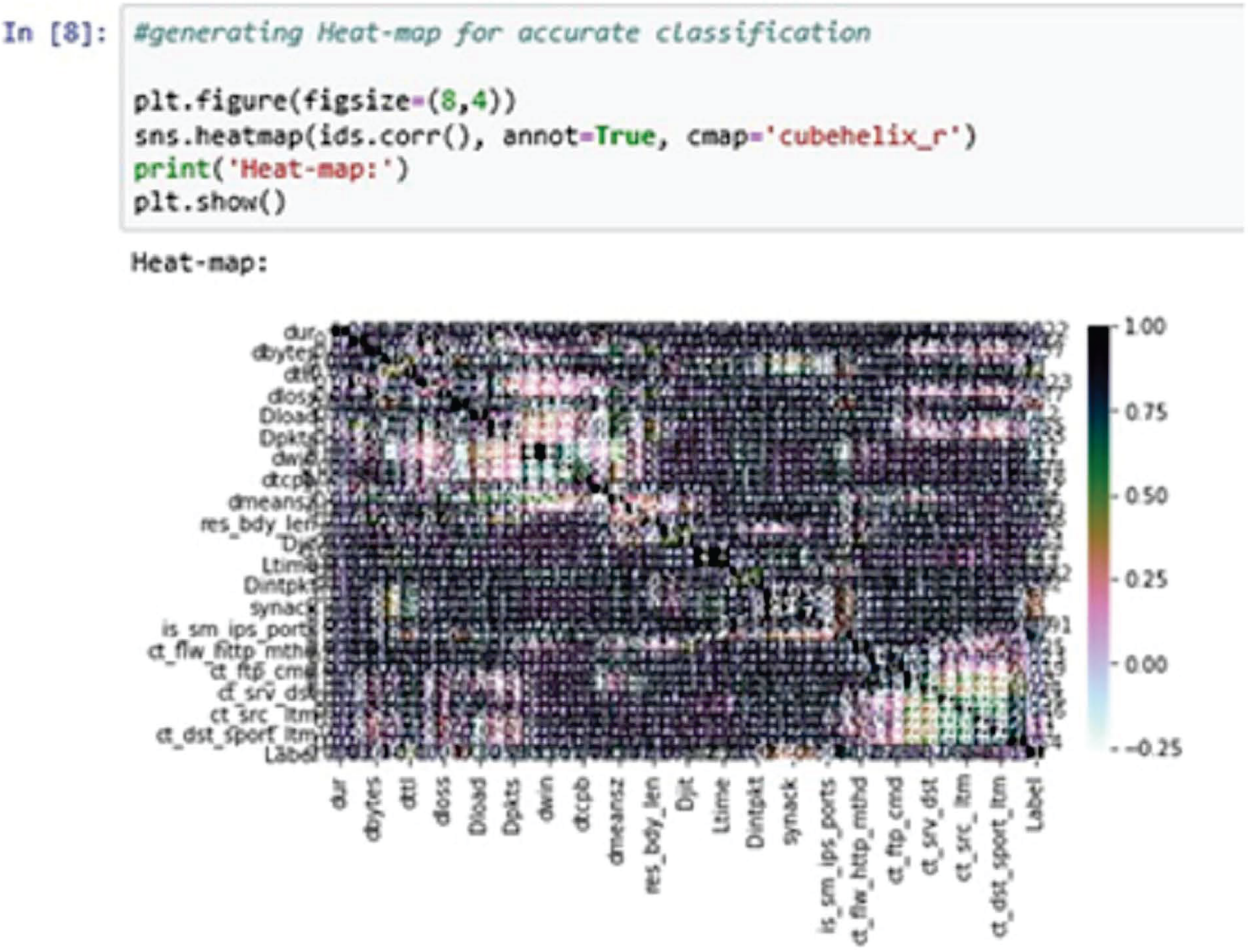
Heat-map.

In the above Fig. 9, the heat-map is used to generate the classification report accurately. Here, we are using the cube helix format to generate the heat-map.

**Figure 10:**
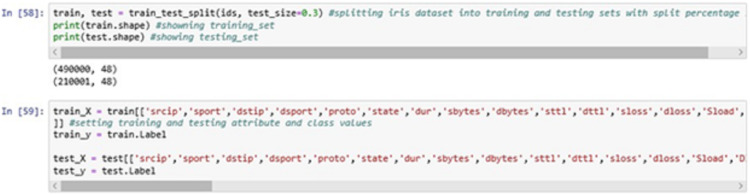
Training and testing phase.

The above Fig. 10 displays the splitting of the dataset into two sets, i.e., training and testing set at 70:30 ration where 70% of the data will be used for the training purpose and remaining 30% of the data will be used for testing purpose i.e., to validate the classification model.

The training and testing are done using the feature set extracted from the dataset entries. The class “Label” is used as the classifier label in this process (Swarna Priya et al., 2020).

**Figure 11:**
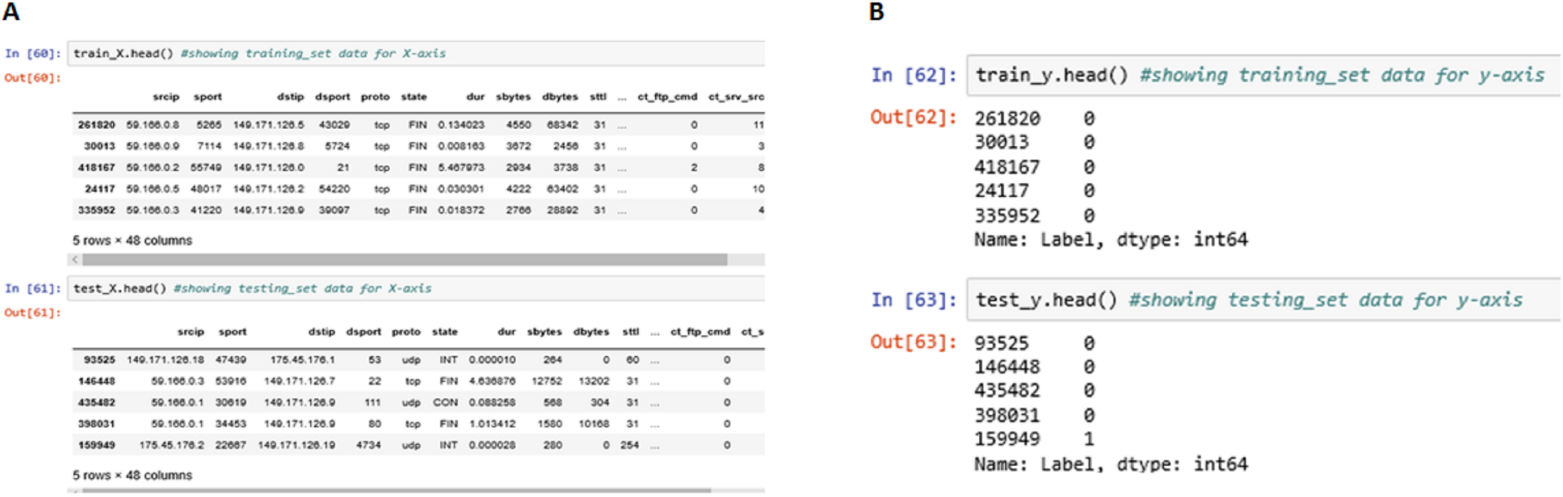
Training and testing information. (A) Training Data Set for the x-axis. (B) Training Data Set for the y-axis.

The above Fig. 11 shows the meta-data stored in train_X, train_y, test_X and test_y partitions of the data entries generated. These four partitions were generated during the training and testing process and were stored in the local repository.

Once the training and testing phase is complete, we applied the three algorithms one by one on the same training set and validated it with the testing set to generate the classification report and confusion matrix.

**Figure 12:**
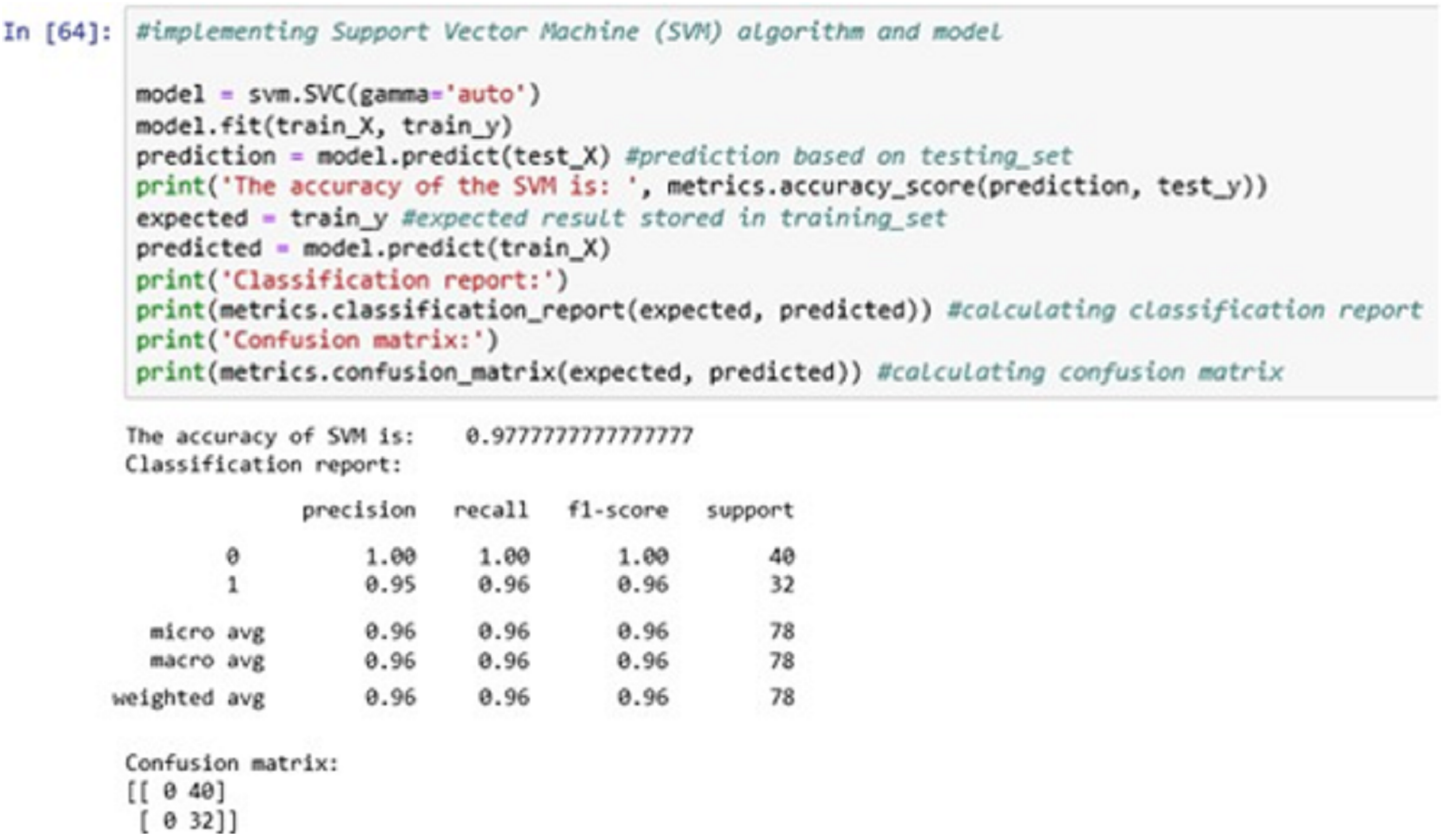
Support vector machine (SVM).

The above Fig. 12 shows the SVM classification model fitting using the SVM algorithm. The accuracy it generates is 97.77777% i.e., the algorithm learns the patterns of the dataset with an accuracy of 97.77777%.

The confusion matrix shows the ratio between the predicted result and the expected results.

**Figure 13:**
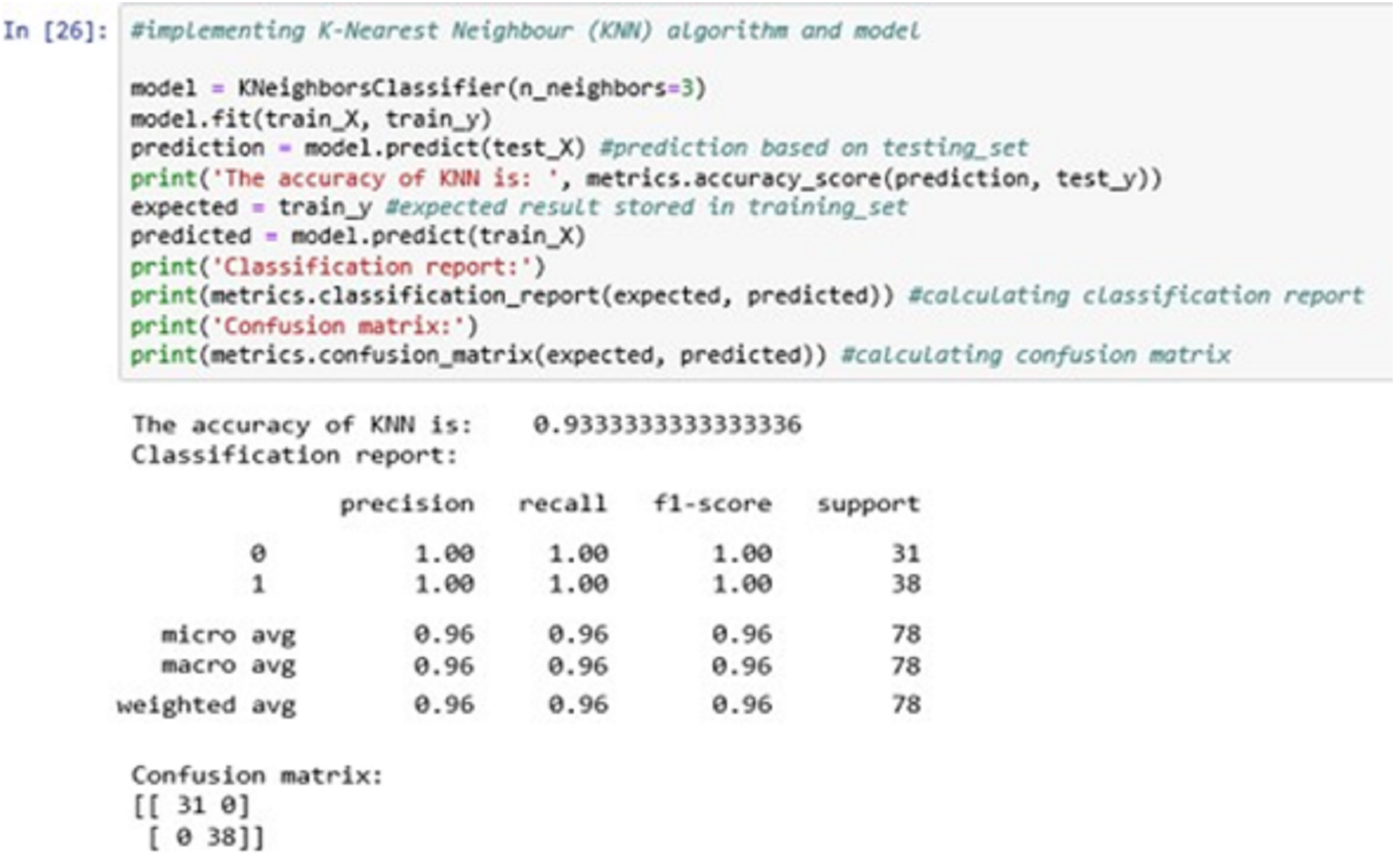
K-nearest neighbour (KNN).

The above Fig. 13 shows the KNN classification model fitting using the KNN algorithm. Here, we are using *k* = 3 for initial experimentations. The value of *k* can be increased or decreased depending on the data entries.

The accuracy it generates is 93.33333% i.e., the algorithm learns the patterns of the dataset with an accuracy of 93.33333%.

**Figure 14:**
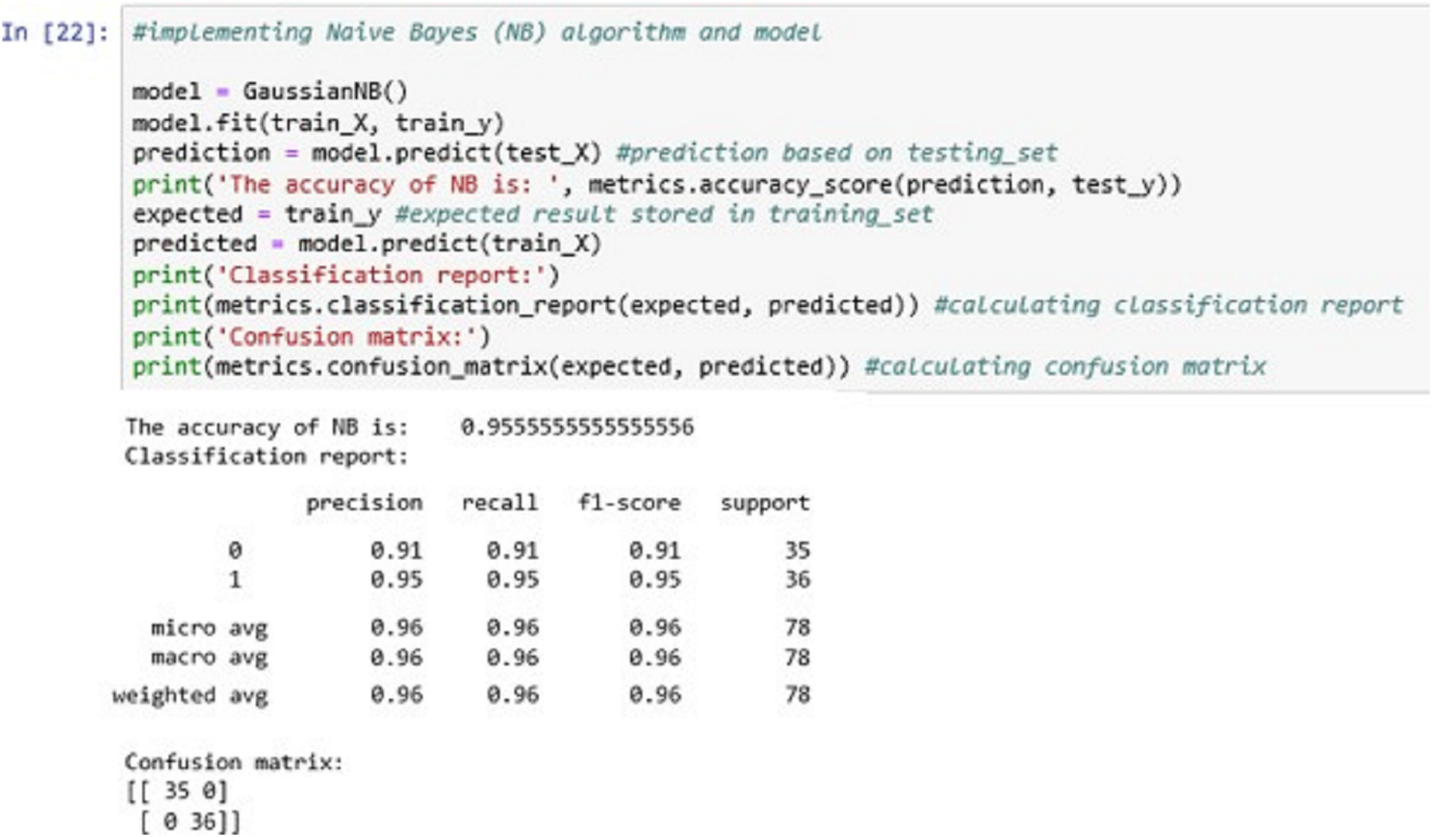
Naïve Bayes (NB).

The above Fig. 14 shows the NB classification model fitting using the NB algorithm. The accuracy it generates is 95.55555% i.e., the algorithm learns the patterns of the dataset with an accuracy of 95.55555%.

By comparing the three algorithms’ accuracy levels, we found that the Support Vector Machine (SVM) has the greatest accuracy level and a better result compared to other two algorithms. Hence, the SVM classification model will be further serialized for future use on any unseen new data (Patgiri et al., 2018).

### Existing model vs. proposed model

This section represents the comparison between the existing model & the proposed model on a different dataset which is INITIAL BCDR-D01 and the results found establishes the conclusion that the proposed model has more accuracy improvement rate (Ernst, Hamed & Kremer, 2018).

The below figures show the implementation result comparison (Accuracy Comparison) between (a) the existing model and (b) the proposed model. The existing model was implemented on the INITIAL BCDR-D01 dataset. This dataset consists of huge collection of data records of patients from all over the world.

To show the proper comparison and the accuracy improvement advantage of our proposed model over the existing model, we have used the same dataset and implemented our model on it.

**Figure 15:**
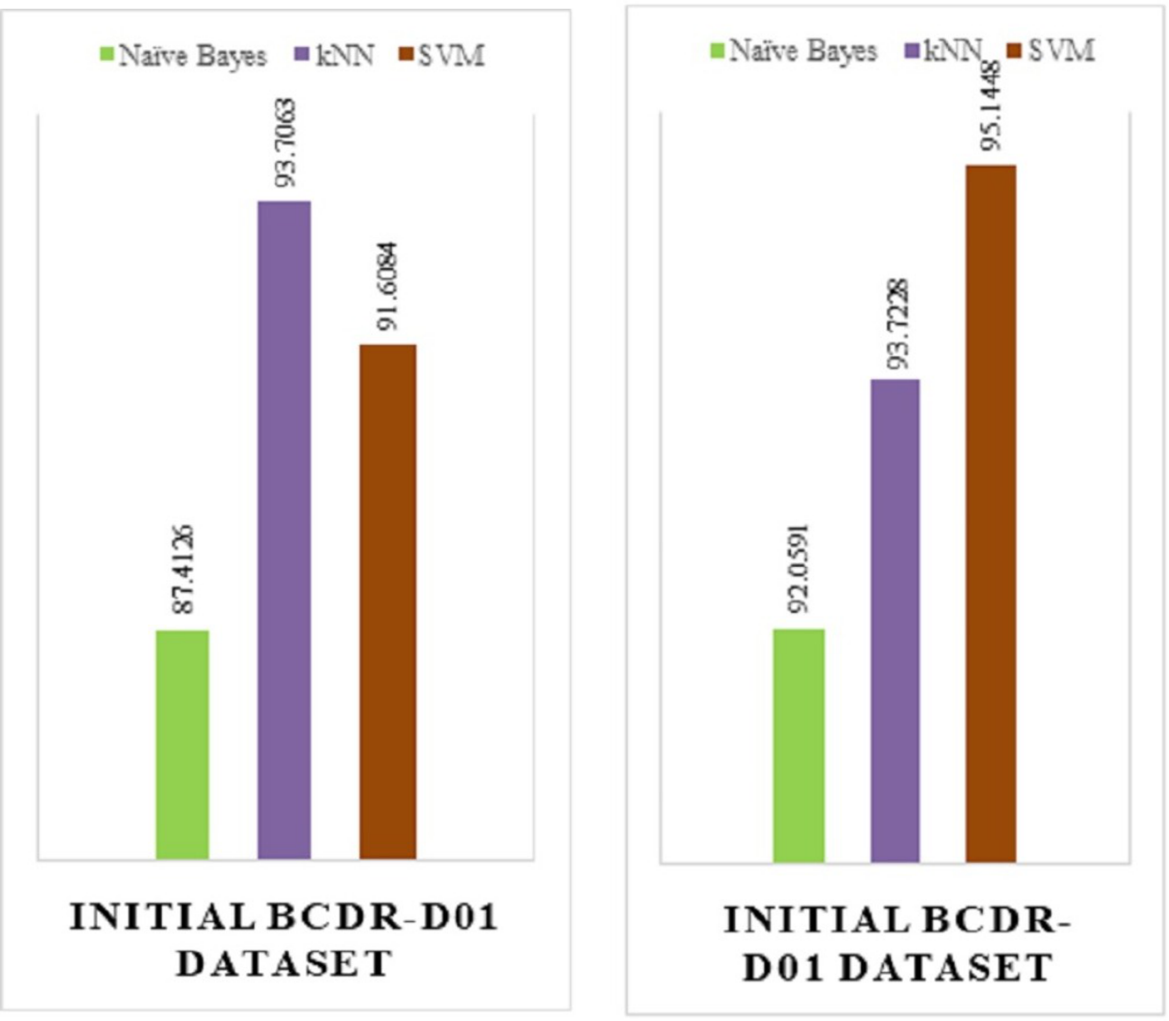
(A) Implementation result of an existing model. (B) Implementation result of existing model.

From the above Fig. 15, we can see that the proposed model (Fig. 15B) has a huge accuracy improvement rate over the existing model (Fig. 15A). The below Table 2 represents the comparison results in a more approachable manner:

**Table 2:** Comparison result between existing and proposed model.

Category	Accuracy of existing model	Accuracy of proposed model
Model name		
Naïve Bayes (NB)	87.4126	92.0591
kNN	93.7063	93.7228
SVM	91.6084	95.1488

From the above table, we can see that for the existing model—Naïve Bayes, kNN and SVM has 87.4126%, 93.706% and 91.6084% of accuracy rate respectively. On the other hand, for the has proposed model—Naïve Bayes, kNN and SVM has 92.0591%, 95.1488% and 93.7228% of accuracy rate respectively, which is a total positive rate of approximately 10% increase in accuracy improvement, which was achieved by fine-tuning the algorithm we have used.

## Results

From the results obtained, we can conclude that Support Vector Machine (SVM) gives better accuracy on the dataset used which is best suitable for aligning with our model. The following thorough investigation and analysis of the results obtained is presented to support our conclusion:

Earlier at the comparison section between existing and proposed model, we found that the proposed model has an approximate accuracy improvement rate of 10% over the existing model in all three algorithms viz Naïve Bayes, kNN and SVM (Moustafa & Slay, 2016). If we narrow down the result obtained further, it can be seen that among the three algorithms used in the proposed model, Support Vector Machine (SVM) has the most improved accuracy rate of approximately 95.1448%.

From the above comparison table and comparison graph, we can see that SVM has the upper hand among all the algorithms used in the proposed model. For ensuring the reliability of the proposed model, we implemented the proposed model on a different dataset (INITIAL BCDR-D01) earlier (Bhattacharya et al., 2020). The results we found supported our conclusion that the algorithm used to implement the SVM model was to-the-point and has an effective accuracy result.

**Figure 16:**
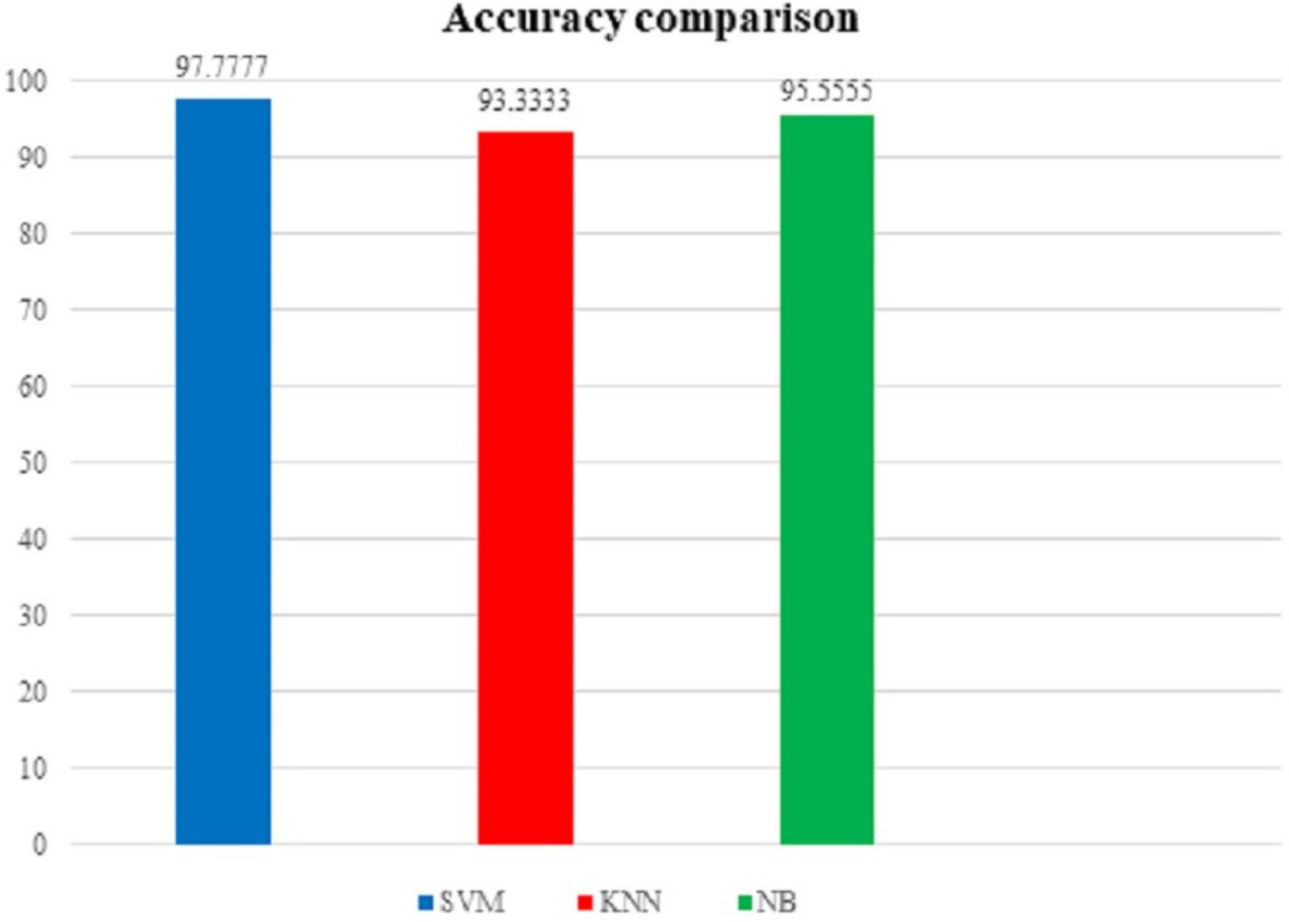
Accuracy comparison graph (using UNSW-NB15 dataset).

To support our findings, below Table 3 shows the comparison table of the results found by implementing the proposed model on our first dataset i.e. the UNSW-NB15 dataset and the Fig. 16 shows the comparison graph prepared by us:

**Table 3:** Accuracy comparison table (using UNSW-NB15 dataset).

Serial No.	Classification algorithms	Accuracy obtained		
1	SVM	97.7777		Highest accuracy obtained
2	kNN	93.3333	⟶	
3	NB	95.5555		

From the above table and figure, we can see that for this case analysis also, SVM has the upper hand among all other algorithms used in the model with approximately 97.7777% accuracy rate.

As a combined result of the scenario 1 (the implementation of proposed model on INITIAL BCDR-D01 dataset) and scenario 2 (the implementation of proposed model on UNSW-NB15 dataset), the statement can be established that SVM (Support Vector Machine) aligns most suitably with our model.

## Conclusion and future prospects

Many techniques have been developed to avoid the network attacks and the intrusions occurring in the network system. As research indicates, the attackers are using the same technology that were developed to protect the cyber-space with an improved capability of a cyber-security breach (Vinayakumar et al., 2019, 2020). Nowadays, gaining access to a remote user’s system without dropping a single malicious file into the system’s mainframe system without dropping a single malicious file into the system’s mainframe is no more impossible.

Researchers suggest that if the defense technologies against the rapidly increasing cyber-threat is not reconsidered, there will be a catastrophically increase in the ratio of cyber-attacks (Kotsiantis, Zaharakis & Pintelas, 2007). As a result, there will be no place in the cyber area where the users’ crucial information will be safe. This raises the question that are we advanced enough to tackle this increased threat and future devastation?

This work focuses on combining the advanced machine learning approaches with the intrusion detection system against different types of intrusion attempts on a network.

It shows the compared result of the accuracy of three classification algorithms on the UNASW-NB15 dataset of the Intrusion Detection System. From the implemented result work obtained, we can conclude that Support Vector Machine (SVM) gives better accuracy on the dataset used, which is best suitable for aligning with our model.

The proposed framework in this research can be further extended to cover the following potentials:

- The trained model can be further used to combine deep learning approaches like Deep Belief Network for further analysis.
- The results can be improved by comparing with other complex machine learning approaches like Random Forest (RF).
- The model can be further extended using the Long Short-Term Memory (LSTM) mechanism to enhance the prediction capability.
- The proposed framework can be applied to the latest released datasets of intrusion detection systems to keep the trained model updated.
- Sample analysis can be done by gathering various malicious programs (malwares, botnets etc.), and the results can be pre-processed and combined with the dataset to create an updated repository which will provide a better accuracy level as the data increases (Zhou et al., 2018).

## Additional Information and Declarations

### Data Availability

The following information was supplied regarding data availability: The code is available at GitHub: https://github.com/amohamed71515/classification-model/blob/main/code.txt. Third-party data is also available at GitHub: https://github.com/naviprem/ids-deep-learning/blob/master/datasets/UNSW-NB15.md.
